# Response of the rhizosphere prokaryotic community of barley (*Hordeum vulgare* L.) to elevated atmospheric CO_2_ concentration in open‐top chambers

**DOI:** 10.1002/mbo3.462

**Published:** 2017-03-30

**Authors:** Márton Szoboszlay, Astrid Näther, Esther Mitterbauer, Jürgen Bender, Hans‐Joachim Weigel, Christoph C. Tebbe

**Affiliations:** ^1^ Thünen Institute of Biodiversity Braunschweig Germany

**Keywords:** 16S rRNA amplicon sequencing, atmospheric CO_2_, Barley, climate change, prokaryotic diversity, rhizosphere

## Abstract

The effect of elevated atmospheric CO_2_ concentration [CO_2_] on the diversity and composition of the prokaryotic community inhabiting the rhizosphere of winter barley (*Hordeum vulgare* L.) was investigated in a field experiment, using open‐top chambers. Rhizosphere samples were collected at anthesis (flowering stage) from six chambers with ambient [CO_2_] (approximately 400 ppm) and six chambers with elevated [CO_2_] (700 ppm). The V4 region of the 16S rRNA gene was PCR‐amplified from the extracted DNA and sequenced on an Illumina MiSeq instrument. Above‐ground plant biomass was not affected by elevated [CO_2_] at anthesis, but plants exposed to elevated [CO_2_] had significantly higher grain yield. The composition of the rhizosphere prokaryotic communities was very similar under ambient and elevated [CO_2_]. The dominant taxa were *Bacteroidetes*,* Actinobacteria*,* Alpha‐*,* Gamma‐*, and *Betaproteobacteria*. Elevated [CO_2_] resulted in lower prokaryotic diversity in the rhizosphere, but did not cause a significant difference in community structure.

## 1 Introduction

Rising atmospheric CO_2_ concentration is an important component of climate change. The globally averaged abundance of atmospheric CO_2_ has increased from 278 ppm in 1750–390.5 ppm in 2011 and is predicted to reach 700 ppm by the end of this century (IPCC, 2013). This change is expected to have strong effects on agroecosystems stimulating plant productivity (Ainsworth & Long, 2005; Ainsworth & Rogers, 2007; He et al., 2012), increasing soil moisture content (He et al., 2012; Hussain et al., 2013) and soil pH (Weigel & Manderscheid, 2012). These effects may alter the activity and composition of the rhizosphere inhabiting microbial community which in turn can greatly affect plant growth and health (Rogers, Runion, & Krupa, 1994). Recent studies investigating the effect of elevated [CO_2_] on the soil or root‐associated prokaryotic diversity or community composition however, reported contradictory results showing either significant (Deng et al., 2012; He et al., 2012; Gschwendtner et al., 2015; Okubo et al., 2015) or no effects (Butterly et al., 2016; Hayden et al., 2012; Ren et al., 2015). It seems that the response of the soil prokaryotic community is characteristic to ecosystems and plant species (Dunbar et al., 2012).

Due to its adaptability and the diversity of its agricultural uses, along with its close evolutionary distance to wheat, barley (*Hordeum vulgare* L.) has often been used as a model plant to study the effects of climate change on the productivity of cereal crops (Dawson et al., 2015). Barley itself is a highly significant crop especially in European agriculture which was responsible for 64.8% of the 144.3 million tons global barley production in 2014 (http://faostat3.fao.org). Barley is stress tolerant, allowing it to grow in cold and/or dry environments. This explains its wide geographic range and often makes it, compared to other cereal crops, more productive under extreme conditions e.g., at high latitudes or under arid climate (Newton et al., 2011; Ullrich, 2011). Despite its agricultural significance and applicability as a model for other cereal crops, there is little information available if climate change might alter the interaction of barley with the soil microbiota. This is particularly true with respect to the response of the rhizosphere prokaryotic community of barley to increasing atmospheric [CO_2_].

In order to assess the implications of elevated [CO_2_] on the prokaryotic diversity and community composition in the rhizosphere of barley, we conducted a field experiment with open‐top chambers. We assumed that increased plant productivity under elevated [CO_2_] would be associated with higher root exudation rate strengthening the rhizosphere effect (Buée, De Boer, Martin, Van Overbeek, & Jurkevitch, 2009). Therefore, we tested the hypothesis that elevated [CO_2_] results in decreased prokaryotic diversity in the rhizosphere of barley.

Recent studies investigating the effect of increased [CO_2_] on the composition of the soil microbial community relied on methods with relatively low resolution, like 16S rRNA gene clone libraries (Dunbar et al., 2012), fingerprinting techniques (Butterly et al., 2016; Gschwendtner et al., 2015), PhyloChip (Hayden et al., 2012; He et al., 2012), and 454 pyrosequencing of 16S rRNA gene amplicons with shallow sequencing depths (Deng et al., 2012 (1698–3299 sequences/sample); Ren et al., 2015 (2,000 sequences/sample); Okubo et al., 2015 (2523–14395 sequences/sample)). However, considering that rare taxa may be more responsive to climate factors, methods with higher resolutions should be applied (Dunbar et al., 2012). Therefore, we used Illumina MiSeq sequencing of 16S rRNA gene amplicons to obtain a magnitude higher resolution of the prokaryotic community composition than previous studies.

## 2 Materials and Methods

**Figure 1:**
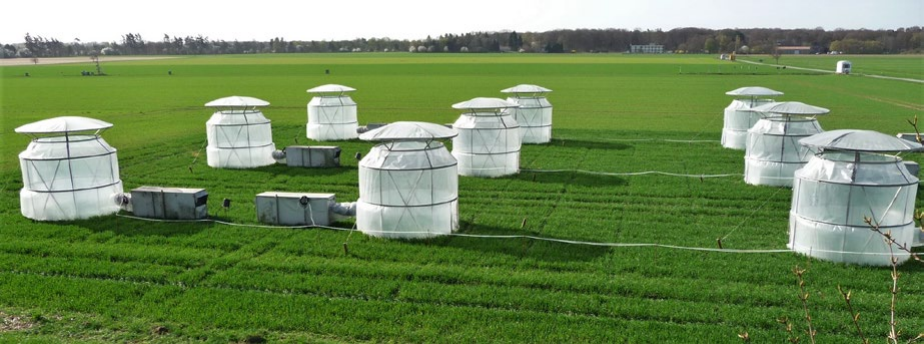
Part of the experimental field showing 9 out of the 12 open‐top chambers

Winter barley (*Hordeum vulgare* L.) cultivar *Kathleen* was grown on an agricultural field in Braunschweig, Germany (52°17′53″N; 10°26′46″E) in 2013. The soil at the site is a loamy sand Luvisol (pH 6.4, organic matter content 1.4% (wt/wt)). Season‐long CO_2_ exposure was carried out in cylindrical open‐top chambers (OTCs) 3.2 m in diameter and 3.0 m high (Figure 1) equipped with CO_2_ dispensing and monitoring systems (Heagle, Philbeck, Rogers, & Letchworth, 1979; Weigel, Adaros, & Jäger, 1987). The atmospheric CO_2_ concentration was elevated to approximately 700 ppm during daylight hours in six OTCs (elevated CO_2_ treatment), while in another six OTCs, the plants were exposed to the ambient, approximately 400 ppm, atmospheric [CO_2_] (ambient CO_2_ treatment). The cultivar *Kathleen* is a six‐row winter barley cultivar with very high grain yield, resistance to mildew, brown rust, and barley yellow mosaic viruses. This cultivar was released in Germany in 2009 (Friedt et al., 2011).

To test the growth response of this cultivar to elevated [CO_2_], we measured shoot dry weight at anthesis and shoot dry weight, the length and dry weight of ears, weight of grains, and number of grains at maturity. Ten plants were collected for these measurements from each OTC. Shoots were dried at 105°C for 16 hr, and ears were dried at 30°C for a week. Grains were cleaned by threshing and winnowing and counted with a Contador seed counter (Pfeuffer, Germany). The data were expressed on a per plant basis, and results from the ambient and elevated CO_2_ treatments were compared with t‐tests in JMP 12.0.1 (SAS Institute, USA).

The rhizoplane with soil particles strongly attached to the root surface were sampled at anthesis for DNA extraction (BBCH65‐69, reached on May 22, 2013 in both the ambient and elevated CO_2_ treatments). In each OTC, three plants were uprooted and their root systems combined. The roots were gently shaken and briefly washed in sterile saline (0.85% NaCl) to remove loosely adhering soil and blotted dry on paper. The roots were then washed in 30 ml sterile saline with 10 rpm for 30 min at 4°C in a rotating shaker (Model 3040, GFL, Burgwedel, Germany) to remove adhering microbial cells. The microbial cells were collected by centrifugation at 4,100*g* for 30 min at 4°C, and the pellets were stored at −80°C. DNA was extracted from the frozen cell pellets, using the FastDNA SPIN kit for soil (MP Biomedicals, Illkirch, France). The extractions included two bead beating steps for 45 s at 6.5 m s^−1^ on a FastPrep‐24 system (MP Biomedicals) and additional washing of the binding matrix with 1 ml 5.5 mol/L guanidine thiocyanate (Carl Roth, Karlsruhe, Germany) if required to recover the original color of the matrix.

The V4 region of the 16S rRNA gene was amplified from the DNA extracts with PCR, using primers S‐D‐Arch‐0519‐a‐S‐15 and S‐D‐Bact‐0785‐a‐A‐21 (Klindworth et al., 2013) modified for paired end sequencing on Illumina MiSeq with the protocol of Kozich, Westcott, Baxter, Highlander, and Schloss (2013). Two PCRs with 50‐μl final volumes were prepared from each DNA extract. Reactions contained 1 μl template, 0.4 μmol/L of each primer, 200 μmol/L of each dNTP, 5% dimethyl sulfoxide, and 2.5 U FastStart High Fidelity Enzyme Blend in 1 x reaction buffer containing 1.8 mmol/L MgCl_2_ (Roche Diagnostics, Mannheim, Germany) (the primer sequences are listed in the supplement). Amplification was carried out in an Applied Biosystems 2720 thermocycler with 2 min initial denaturation at 95°C followed by 35 cycles of 95°C for 30 s, 50°C for 30 s, and 72°C for 1 min, and a final extension at 72°C for 5 min. Products from the two replicate reactions were pooled and purified from agarose gels with a HiYield PCR Clean‐up & Gel‐Extraction kit (SLG) and quantified with Quant‐iT PicoGreen dsDNA assay (Invitrogen, Darmstadt, Germany), using a Mithras LB 940 microplate reader (Berthold Technologies, Bad Wildbad, Germany). Equimolar amounts from all purified PCR products were pooled and sent to StarSEQ (Mainz, Germany) for sequencing on an Illumina MiSeq instrument with a 500 cycle v2 kit (Illumina, San Diego, Ca) according to the protocol of Kozich et al., 2013. Sequences were deposited at the European Nucleotide Archive (http://www.ebi.ac.uk/ena) under accession number PRJEB15090.

Sequences were processed with DADA2 version 1.1.1 (Callahan et al., 2016). DADA2 does not group the sequences into operational taxonomic units, instead it groups sequences together that are likely to originate from the same parent sequence variant (SV) and only differ due to sequencing errors. Based on the quality scores, the first 10 bases of the sequences were trimmed, the forward reads were truncated at position 240, and the reverse reads were truncated at position 160. Reads with maximum number of expected errors over two, and reads containing ambiguous bases or a quality score of two were discarded. The DADA algorithm was run with the pool option. Forward and reverse reads were then merged, chimeric sequences were removed, and the resulting sequence variants (SVs) were classified based on the SILVA reference release 123 (Pruesse et al., 2007). Only taxonomic assignments with 70% or higher bootstrap support were accepted. SVs classified as mitochondrial, chloroplast, or eukaryotic sequences, as well as SVs not possible to classify at the domain level with 70% or a higher bootstrap support were deleted from the dataset.

We did not subsample the dataset (McMurdie & Holmes, 2014). Instead, relativization or centered log‐ratio transformation (Gloor, Wu, Pawlowsky‐Glahn, & Egozcue, 2016) was applied when needed to correct for differences in sequencing depth. Shannon and Simpson diversity indices were calculated in R 3.3.1 (www.R-project.org), using the vegan package version 2.4‐0 (Oksanen et al., 2016) after relativizing the dataset, and results from the ambient and elevated CO_2_ treatments were compared with two‐tailed t‐tests in JMP 12.0.1. ANOSIM was used to compare the structure of the prokaryotic communities in the two treatments. For this analysis, the dataset was not relativized, but rare SVs containing less than 91 sequences (this corresponds to 0.01% relative abundance) were removed. The resulting data matrix contained 1,074 SVs encompassing 91.1% of the sequences in the original data matrix. All zeroes in the data matrix (3.15% of the matrix) were then replaced with 0.1 to allow applying centered log‐ratio transformation to the dataset, using the compositions package version 1.40.1 in R (Van den Boogaart & Tolosana‐Delgado, 2008). ANOSIM (vegan package) was run with the resulting data matrix, using Euclidean distances in R.

Using the complete, nonrelativized data matrix, ALDEx2 version 1.4.0 (Fernandes et al., 2014) was applied in R with 1,000 Monte Carlo samples and Welch's t‐test to identify SVs with different abundance in the two treatments. SVs were grouped according to their taxonomic classification from genus to phylum level, and the ALDEx2 analysis was repeated to test taxa for differential abundance between the two treatments. ALDEx2 results were corrected for multiple testing with 10% target false discovery rate.

To analyze SVs shared by most samples from both treatments and SVs only detectable in samples from one treatment, the dataset was first rarefied to contain 59,400 sequences in every sample and converted into biom format in mothur version 1.37.4 (Schloss et al., 2009). SV‐sharing networks were calculated in QIIME virtual box version 1.9.1 (Caporaso et al., 2010) and visualized in Cytoscape 2.8.3 (http://www.cytoscape.org) with edge‐weighted spring embedded layout. Scripts used to run the analysis are listed in the supplement.

## 3 Results

There was no statistically significant difference in shoot dry weight between the plants from the elevated and ambient CO_2_ treatments neither at anthesis nor at maturity (data not shown). However, at maturity, plants grown under the elevated CO_2_ treatment had significantly higher grain yield (grain weight per plant) which seems to be attributed to a higher number of grains per ear (Table 1).

**Table 1:** Plant yield parameters (average ± SD) with *p*‐values from two‐tailed t‐tests

	Ambient CO_2_ treatment	Elevated CO_2_ treatment	*p*‐value
Ear length (cm)	6.3 ± 0.80	7.4 ± 0.34	0.028
Ear dry weight (g)	11.2 ± 1.82	13.7 ± 1.67	0.047
Grain weight (g/plant)	10.0 ± 1.59	12.4 ± 1.50	0.037
Grain number (/plant)	160 ± 25.0	192 ± 20.2	0.030

We obtained in total 1,512,086 sequences from the samples. After removing low quality, chimeric, mitochondrial, and chloroplast sequences, and sequences that could not be classified as bacterial or archaeal, 55.6%–64.3% of the sequences were retained in the samples (910,958 sequences in total) forming 3,618 SVs. The length of the SVs ranged between 230 and 253 nucleotides with 90.4% of them being 231 bp long. The individual samples contained 59,400–90,691 sequences and 2,288–2,931 SVs. Only 21 SVs were classified as *Archaea* containing a total of 7,265 sequences across the entire dataset. All of them fell into the phylum *Thaumarchaeota*. The bacterial sequences were classified into 28 phyla (Table S1). Prokaryotic communities in the samples were dominated by *Proteobacteria* (44.8%–55% of the sequences in a sample), *Bacteriodetes* (13.1%–30.6%), and *Actinobacteria* (9.8%–25.3%). Within *Proteobacteria*,* Alphaproteobacteria* (14.6%–21.3% of all sequences in a sample), *Gammaproteobacteria* (13.2%–24.6%), and *Betaproteobacteria* (7.5%–15.9%) were the most abundant.

**Figure 2:**
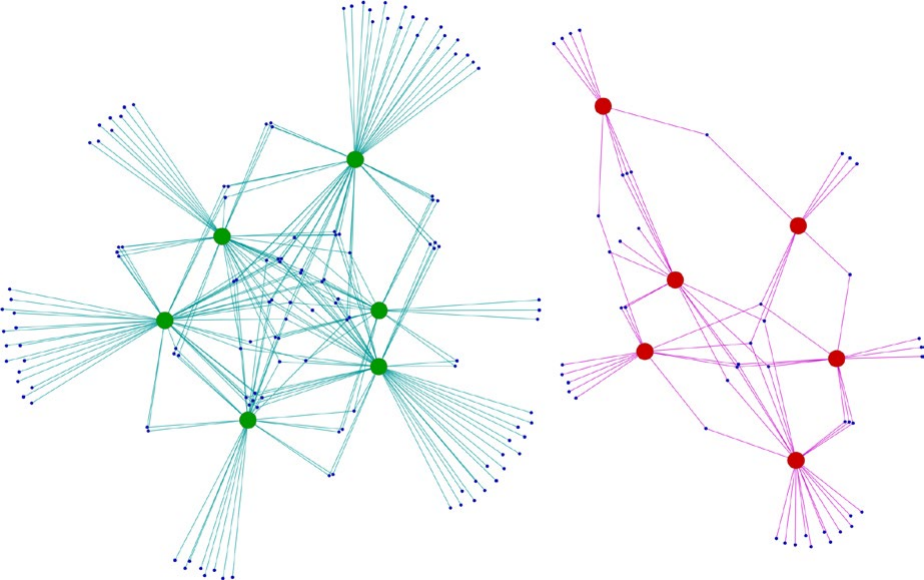
Sequence variant (SV)‐sharing networks of SVs only detected in samples from the ambient CO_2_ treatment (left), or in samples from the elevated CO_2_ treatment (right). Edge‐weighted spring embedded layout. Large green and red nodes represent samples, small blue nodes represent SVs

The samples from the ambient CO_2_ treatment had a Shannon diversity of 6.231 ± 0.278 (average ± standard deviation) and a Simpson diversity of 0.9934 ± 0.0019. The Shannon diversity of the samples from the elevated CO_2_ treatment was 5.804 ± 0.340 and their Simpson diversity was 0.9880 ± 0.0044. The difference in the rhizosphere prokaryotic diversity between the two treatments was therefore on average 0.427 in Shannon diversity (*p *= 0.056), and 0.0054 in Simpson diversity (*p *= 0.041). There was no significant difference in community structure between the samples from the two treatments (ANOSIM R = −0.024, *p *= 0.568). Furthermore, no SVs were identified with ALDEx2 to have significantly different abundance in the samples from the elevated CO_2_ treatment than in the samples from the ambient CO_2_ treatment. SVs were grouped according to their taxonomic classification, but ALDEx2 found no genera, families, orders, classes, or phyla with significantly different abundance between the treatments. The composition of the prokaryotic communities was very similar for both treatments: 2,249 out of the 3,618 SVs were detected in at least four out of the six samples in both treatments, and these SVs included 96.1% of the sequences in the whole dataset. There were, however, SVs only detectable in samples from one of the treatments (Figure 2). These were not identified by ALDEx2 as being significantly differentially abundant between treatments either because they were present in only a few samples and not in most of the samples from a treatment, or because their abundances were low. We found 135 SVs (3.7% of all SVs) only detectable in samples from the ambient CO_2_ treatment, but only 48 SVs (1.3%) unique to samples from the elevated CO_2_ treatment. These SVs contained 802 (0.09%) and 392 sequences (0.04%), respectively, and were classified into a wide range of bacterial taxa (Table S2).

## 4 Discussion

We found that the rhizosphere prokaryotic community of barley was dominated by *Proteobacteria*,* Bacteroidetes*, and *Actinobacteria*. The dominance of these three phyla in the barley rhizosphere was confirmed by other studies (Bulgarelli et al., 2015; Pii et al., 2016). In an investigation of the rhizosphere bacterial communities of different barley varieties, these three phyla were found to be as abundant (88% average relative abundance) as in our results (90% average relative abundance, Table S1) (Bulgarelli et al., 2015). The dominance of *Proteobacteria* and *Actinobacteria* on the root surface of barley was confirmed by microscopic analyses, using fluorescent in situ hybridization (Spohn, Treichel, Cormann, Schloter, & Fischer, 2015).

The composition of the rhizosphere prokaryotic communities was very similar under ambient and elevated [CO_2_], and SVs detectable just in samples from one of the treatments were only a small fraction of the rhizosphere prokaryotic communities. However, the SVs unique to the ambient CO_2_ treatment were more abundant and diverse than the SVs only detected in the elevated CO_2_ treatment (Figure 2). Another study, using PhyloChips also found only a minor fraction of the soil bacterial community to be detectable only under elevated or only under ambient atmospheric CO_2_ concentration with more phylotypes being unique to the ambient CO_2_ treatment (He et al., 2012). In our study, SVs detectable only in the ambient or elevated CO_2_ treatments represent 16 and 10 phyla, respectively (Table S2). Due to this large diversity and their low abundance, it is currently difficult to assess their ecological significance.

According to our expectations, higher [CO_2_] resulted in increased productivity of barley and a decrease of the prokaryotic diversity in its rhizosphere. Higher [CO_2_] may impact the prokaryotic diversity in the rhizosphere by several means: (1) Increased plant productivity results in higher rhizodeposition rate (Cheng, 1999). This means more nutrient input into the rhizosphere that selects for microorganisms that can efficiently grow on these plant‐derived compounds. (2) Increasing atmospheric CO_2_ concentration increases the nitrogen uptake of plants that can cause nitrogen limitation in the rhizosphere (Hu, Chapin, Firestone, Field, & Chiariello, 2001). (3) The soil moisture content can increase due to lower plant evapotranspiration under high [CO_2_] (He et al., 2012; Hussain et al., 2013). All of these factors may also affect soil pH. In our experiment, a slow‐release fertilizer supplying 160 kg ha^−1^ nitrogen was applied to the field and the plants showed no symptoms of nutrient deficiency. Therefore, it is unlikely that nitrogen limitation in the elevated CO_2_ treatment caused the decrease in the rhizosphere prokaryotic diversity. Soil moisture content was regularly measured in the OTCs and irrigation was applied to correct for differences. Thus, the most likely explanation for the decrease of the rhizosphere prokaryotic diversity should be altered rhizodeposition under elevated [CO_2_].

Recent studies either reported a decrease in bacterial diversity under elevated [CO_2_] (Gschwendtner et al., 2015; Okubo et al., 2015) or found no effect (Deng et al., 2012; Hayden et al., 2012; Ren et al., 2015). This controversy in the literature may not only be due to differences between the studied ecosystems or the resolution of the applied methods but, in part, can derive from the sampling technique. Many studies sampled soil cores taken from the surface 5–15 cm at random locations (Butterly et al., 2016; Deng et al., 2012; Dunbar et al., 2012; Hayden et al., 2012; He et al., 2012; Ren et al., 2015) which may contain soil very strongly influenced by the roots and also soil further away from roots in undefined proportions. In contrast, we sampled the rhizoplane and soil particles most closely attached to the root surface. This may be a very different community than what is obtained by mixing soil cores. For example, using fluorescent in situ hybridization, it was shown that bacterial abundance differs at a very small spatial scale in the rhizosphere of barley (Spohn et al., 2015). It was significantly higher at the root surface than at a 30 μm distance. An investigation of the rhizosphere bacterial community of young beech trees demonstrated that exposure to high [CO_2_] only affected the communities associated with fine roots and tightly adhering soil, but not the rhizosphere soil fraction that was less closely attached to the roots (Gschwendtner et al., 2015). It is noteworthy that the other two recent studies that, similarly to ours, looked at communities closely associated with the roots (Gschwendtner et al., 2015; Okubo et al., 2015) also found significantly decreased bacterial diversity under elevated atmospheric [CO_2_], although they investigated different plant species (beech trees and rice) in different soils growing under different environmental conditions. Elevated atmospheric [CO_2_] was found to decrease the Shannon diversity of the root‐associated bacterial communities of young beech trees on average by 0.4 (Gschwendtner et al., 2015) which is similar to our results (0.427). In the case of rice, the change in Shannon diversity due to increased atmospheric [CO_2_] was similar to our results with some rice cultivars (0.589, 0.674), but substantially smaller (0.109), or larger (1.271) changes were found with other cultivars, and the change in Simpson diversity was over 10‐fold higher with most rice cultivars than in our study (Okubo et al., 2015 and personal communication). It has to be noted, that differences in the methods, for example in the sequencing depth, can influence diversity index values. However, the decrease of diversity is probably a common response of the root‐associated bacterial community triggered by elevated CO_2_ and mediated through the plants into the soil ecosystem, but the size of this response appears to vary between plant species and cultivars.

## Conflict of Interest

None declared.

## References

[mbo3462-bib-0001] Ainsworth, E. A. , & Long, S. P. (2005). What have we learned from 15 years of free‐air CO_2_ enrichment (FACE)? A meta‐analytic review of the responses of photosynthesis, canopy. New Phytologist, 165, 351–371. 15720649 10.1111/j.1469-8137.2004.01224.x

[mbo3462-bib-0002] Ainsworth, E. A. , & Rogers, A. (2007). The response of photosynthesis and stomatal conductance to rising [CO_2_]: Mechanisms and environmental interactions. Plant, Cell and Environment, 30, 258–270. 10.1111/j.1365-3040.2007.01641.x 17263773

[mbo3462-bib-0003] Buée, M. , De Boer, W. , Martin, F. , Van Overbeek, L. , & Jurkevitch, E. (2009). The rhizosphere zoo: An overview of plant‐associated communities of microorganisms, including phages, bacteria, archaea, and fungi, and of some of their structuring factors. Plant and Soil, 321, 189–212.

[mbo3462-bib-0004] Bulgarelli, D. , Garrido‐Oter, R. , Münch, P. C. , Weiman, A. , Dröge, J. , Pan, Y. , … Schulze‐Lefert, P . (2015). Structure and function of the bacterial root microbiota in wild and domesticated barley. Cell Host & Microbe, 17, 392–403. 25732064 10.1016/j.chom.2015.01.011 PMC4362959

[mbo3462-bib-0005] Butterly, C. R. , Phillips, L. A. , Wiltshire, J. L. , Franks, A. E. , Armstrong, R. D. , Chen, D. , … Tang, C . (2016). Long‐term effects of elevated CO_2_ on carbon and nitrogen functional capacity of microbial communities in three contrasting soils. Soil Biology & Biochemistry, 97, 157–167.

[mbo3462-bib-0006] Callahan, B. J. , McMurdie, P. J. , Rosen, M. J. , Han, A. W. , Johnson, A. J. , & Holmes, S. P. (2016). DADA2: High resolution sample inference from amplicon data. Nature Methods, 13, 581–583. 27214047 10.1038/nmeth.3869 PMC4927377

[mbo3462-bib-0007] Caporaso, J. G. , Kuczynski, J. , Stombaugh, J. , Bittinger, K. , Bushman, F. D. , Costello, E. K. , … Knight, R. (2010). QIIME allows analysis of high‐throughput community sequencing data. Nature Methods, 7, 335–336. 20383131 10.1038/nmeth.f.303 PMC3156573

[mbo3462-bib-0008] Cheng, W. (1999). Rhizosphere feedbacks in elevated CO_2_ . Tree Physiology, 19, 313–320. 12651574 10.1093/treephys/19.4-5.313

[mbo3462-bib-0009] Dawson, I. K. , Russell, J. , Powell, W. , Steffenson, B. , Thomas, W. T. , & Waugh, R. (2015). Barley: A translational model for adaptation to climate change. New Phytologist, 206, 913–931. 25605349 10.1111/nph.13266

[mbo3462-bib-0010] Deng, Y. , He, Z. L. , Xu, M. Y. , Qin, Y. J. , Van Nostrand, J. D. , Wu, L. Y. , et al. (2012). Elevated carbon dioxide alters the structure of soil microbial communities. Applied and Environmental Microbiology, 78, 2991–2995. 22307288 10.1128/AEM.06924-11 PMC3318805

[mbo3462-bib-0011] Dunbar, J. , Eichorst, S. A. , Gallegos‐Graves, L. , Silva, S. , Xie, G. , Hengartner, N. W. , … Kuske, C. R . (2012). Common bacterial responses in six ecosystems exposed to 10 years of elevated atmospheric carbon dioxide. Environmental Microbiology, 14, 1145–1158. 22264231 10.1111/j.1462-2920.2011.02695.x

[mbo3462-bib-0012] Fernandes, A. D. , Reid, J. N. , Macklaim, J. M. , McMurrough, T. A. , Edgell, D. R. , … Gloor, G. B. (2014). Unifying the analysis of high‐throughput sequencing datasets: Characterizing RNA‐seq, 16S rRNA gene sequencing and selective growth experiments by compositional data analysis. Microbiome, 2, 15. 24910773 10.1186/2049-2618-2-15 PMC4030730

[mbo3462-bib-0013] Friedt, W. , Horsley, R. D. , Harvey, B. L. , Poulsen, D. M. , Lance, R. , Ceccarelli, S. , … Capettini F. (2011). Barley breeding history, progress, objectives, and technology In Ullrich S. E. (Ed.), Barley: Production, Improvement, and Uses (pp. 160–220). Oxford, UK: Blackwell Publishing Ltd.

[mbo3462-bib-0014] Gloor, G. B. , Wu, J. R. , Pawlowsky‐Glahn, V. , & Egozcue, J. J. (2016). It's all relative: Analyzing microbiome data as compositions. Annals of Epidemiology, 26, 322–329. 27143475 10.1016/j.annepidem.2016.03.003

[mbo3462-bib-0015] Gschwendtner, S. , Leberecht, M. , Engel, M. , Kublik, S. , Dannenmann, M. , Polle, A. , & Schloter, M. (2015). Effects of elevated atmospheric CO_2_ on microbial community structure at the plant‐soil interface of young beech trees (*Fagus sylvatica* L.) grown at two sites with contrasting climatic conditions. Microbial Ecology, 69, 867–878. 25370887 10.1007/s00248-014-0527-x

[mbo3462-bib-0016] Hayden, H. L. , Mele, P. M. , Bougoure, D. S. , Allan, C. Y. , Norng, S. , Piceno, Y. M. , … Hovenden, M. J. (2012). Changes in the microbial community structure of bacteria, archaea and fungi in response to elevated CO_2_ and warming in an Australian native grassland soil. Environmental Microbiology, 14, 3081–3096. 23039205 10.1111/j.1462-2920.2012.02855.x

[mbo3462-bib-0017] He, Z. L. , Piceno, Y. , Deng, Y. , Xu, M. Y. , Lu, Z. M. , DeSantis, T. , … Zhou, J . (2012). The phylogenetic composition and structure of soil microbial communities shifts in response to elevated carbon dioxide. ISME Journal, 6, 259–272. 21796217 10.1038/ismej.2011.99 PMC3260516

[mbo3462-bib-0018] Heagle, A. S. , Philbeck, R. B. , Rogers, H. H. , & Letchworth, M. B. (1979). Dispensing and monitoring ozone in open top field chambers for plant effect studies. Phytopathology, 69, 15–20.

[mbo3462-bib-0019] Hu, S. , Chapin, F. S. , Firestone, M. K. , Field, C. B. , & Chiariello, N. R. (2001). Nitrogen limitation of microbial decomposition in a grassland under elevated CO_2_ . Nature, 409, 188–191. 11196641 10.1038/35051576

[mbo3462-bib-0020] Hussain, M. Z. , Vanloocke, A. , Siebers, M. H. , Ruiz‐Vera, U. M. , Markelz, R. J. C. , Leaky, A. D. B. , … Bernacchi, C. J . (2013). Future carbon dioxide concentration decreases canopy evapotranspiration and soil water depletion by field‐gown maize. Global Change Biology, 19, 1574–1584. 10.1111/gcb.12155 23505040

[mbo3462-bib-0021] IPCC (2013). Climate Change 2013: The Physical Science Basis. Cambridge, UK: Cambridge University Press.

[mbo3462-bib-0022] Klindworth, A. , Pruesse, E. , Schweer, T. , Peplies, J. , Quast, C. , Horn, M. , & Glöckner, F. O . (2013). Evaluation of general 16S ribosomal RNA gene PCR primers for classical and next‐generation sequencing‐based diversity studies. Nucleic Acids Research, 41, e1. 22933715 10.1093/nar/gks808 PMC3592464

[mbo3462-bib-0023] Kozich, J. J. , Westcott, S. L. , Baxter, N. T. , Highlander, S. K. , & Schloss, P. D. (2013). Development of a dual‐index sequencing strategy and curation pipeline for analyzing amplicon sequence data on the MiSeq Illumina sequencing platform. Applied and Environment Microbiology, 79, 5112–5120. 10.1128/AEM.01043-13 PMC3753973 23793624

[mbo3462-bib-0024] McMurdie, P. J. , & Holmes, S. (2014). Waste not, want not: Why rarefying microbiome data is inadmissible. PLoS Computational Biology, 10, e1003531. 24699258 10.1371/journal.pcbi.1003531 PMC3974642

[mbo3462-bib-0025] Newton, A. C. , Flavell, A. J. , George, T. S. , Leat, P. , Mullholland, B. , Ramsay, L. , … Bingham, I. J . (2011). Crops that feed the world 4. Barley: A resilient crop? Strengths and weaknesses in the context of food security. Food Security, 3, 141–178.

[mbo3462-bib-0026] Oksanen, J. , Blanchet, F. G. , Friendly, M. , Kindt, R. , Legendre, P. , McGlinn, D. , Wagner, H . (2016). Vegan: community ecology package. R package version 2.4‐0. https://CRAN.R-project.org/package=vegan

[mbo3462-bib-0027] Okubo, T. , Liu, D. , Tsurumaru, H. , Ikeda, S. , Asakawa, S. , Tokida, T. , et al. (2015). Elevated atmospheric CO_2_ levels affect community structure of rice root‐associated bacteria. Frontiers in Microbiology, 6, 136. 25750640 10.3389/fmicb.2015.00136 PMC4335179

[mbo3462-bib-0028] Pii, Y. , Borruso, L. , Brusetti, L. , Crecchio, C. , Cesco, S. , & Mimmo, T. (2016). The interaction between iron nutrition, plant species and soil type shapes the rhizosphere microbiome. Plant Physiology and Biochemistry, 99, 39–48. 26713550 10.1016/j.plaphy.2015.12.002

[mbo3462-bib-0029] Pruesse, E. , Quast, C. , Knittel, K. , Fuchs, B. M. , Ludwig, W. , Peplies, J. , & Glöckner, F. O. (2007). SILVA: A comprehensive online resource for quality checked and aligned ribosomal RNA sequence data compatible with ARB. Nucleic Acids Research, 35, 7188–7196. 17947321 10.1093/nar/gkm864 PMC2175337

[mbo3462-bib-0030] Ren, G. , Zhu, C. , Alam, M. S. , Tokida, T. , Sakai, H. , Nakamura, H. , … Jia, Z . (2015). Response of soil, leaf endosphere and phyllosphere bacterial communities to elevated CO_2_ and soil temperature in a rice paddy. Plant and Soil, 392, 27–44.

[mbo3462-bib-0031] Rogers, H. H. , Runion, G. B. , & Krupa, S. V. (1994). Plant‐responses to atmospheric CO_2_ enrichment with emphasis on roots and the rhizosphere. Environmental Pollution, 83, 155–189. 15091762 10.1016/0269-7491(94)90034-5

[mbo3462-bib-0032] Schloss, P. D. , Westcott, S. L. , Ryabin, T. , Hall, J. R. , Hartmann, M. , Hollister, E. B. , … Weber, C. F . (2009). Introducing mothur: Open‐source, platform‐independent, community‐supported software for describing and comparing microbial communities. Applied and Environmental Microbiology, 75, 7537–7541. 19801464 10.1128/AEM.01541-09 PMC2786419

[mbo3462-bib-0033] Spohn, M. , Treichel, N. S. , Cormann, M. , Schloter, M. , & Fischer, D. (2015). Distribution of phosphatase activity and various bacterial phyla in the rhizosphere of *Hordeum vulgare* L. depending on P availability. Soil Biology & Biochemistry, 89, 44–51.

[mbo3462-bib-0034] Ullrich, S. E . (2011). Significance, adaptation, production, and trade of barley In S. E Ullrich (Eds.), Barley: Production, improvement, and uses (pp. 3–13). Oxford, UK: Blackwell Publishing Ltd.

[mbo3462-bib-0035] Van den Boogaart, K. G. , & Tolosana‐Delgado, R. (2008). “Compositions”: A unified R package to analyze compositional data. Computers & Geosciences, 34, 320–338.

[mbo3462-bib-0036] Weigel, H. J. , Adaros, G. , & Jäger, H. J. (1987). An open‐top chamber study with filtered and non‐filtered air to evaluate the effects of air pollutants on crops. Environmental Pollution, 47, 231–244. 15092710 10.1016/0269-7491(87)90213-2

[mbo3462-bib-0037] Weigel, H. J. , & Manderscheid, R. (2012). Crop growth responses to free air CO_2_ enrichment and nitrogen fertilization: Rotating barley, ryegrass, sugar beet and wheat. European Journal of Agronomy, 43, 97–107.

