# Supplementary material for: Response of the rhizosphere prokaryotic community of barley (Hordeum vulgare L.) to elevated atmospheric CO2 concentration in open‐top chambers
Source: Microbiologyopen. 2017 Mar 30;6(4):e00462. doi: 10.1002/mbo3.462 (PMC5552935; doi:10.1002/mbo3.462)
Supplement: Supplementary file 1 [file MBO3-6-na-s001.pdf]

Table S1: Average relative abundance (%) of archaeal and bacterial phyla detected in the soil samples

Sequence variants that were identified as bacterial, but could not be classified to any phylum with at least 70% bootstrap support are collected in the 'unclassified' group.

Proteobacteria	50.1690
Bacteroidetes	21.0102
Actinobacteria	18.4299
Verrucomicrobia	2.0950
Acidobacteria	1.8830
Firmicutes	1.5448
Chloroflexi	1.3038
Gemmatimonadetes	0.8021
Thaumarchaeota	0.7909
Planctomycetes	0.7453
Fibrobacteres	0.4391
unclassified	0.2227
Armatimonadetes	0.1751
Nitrospirae	0.1610
Cyanobacteria	0.0845
Latescibacteria	0.0425
Chlorobi	0.0225
TM6	0.0196
JL-ETNP-Z39	0.0163
Elusimicrobia	0.0124
SHA-109	0.0094
WCHB1-60	0.0075
Hydrogenedentes	0.0056
Deinococcus-Thermus	0.0022
Spirochaetae	0.0020
Tenericutes	0.0016
Saccharibacteria	0.0008
WD272	0.0004
Chlamydiae	0.0004
SM2F11	0.0003
