# Supplementary material for: Response of the rhizosphere prokaryotic community of barley (Hordeum vulgare L.) to elevated atmospheric CO2 concentration in open‐top chambers
Source: Microbiologyopen. 2017 Mar 30;6(4):e00462. doi: 10.1002/mbo3.462 (PMC5552935; doi:10.1002/mbo3.462)
Supplement: Supplementary file 3 [file MBO3-6-na-s003.docx]

Response of the rhizosphere microbial community of barley (*Hordeum vulgare* L.) to elevated atmospheric CO_2_ concentration in open-top chambers – Supplement

Primer sequences:

Data processing and statistical analysis

Processing the sequences with DADA2 in R:

library(dada2); packageVersion("dada2")

# [1] ‘1.1.1’

library(ShortRead); packageVersion("ShortRead")

# [1] ‘1.30.0’

library(ggplot2); packageVersion("ggplot2")

# [1] ‘2.1.0’

path <- "path to the work directory"

fns <- list.files(path)

fastqs <- fns[grepl(".fastq", fns)]

fastqs <- sort(fastqs)

fnFs <- fastqs[grepl("_R1", fastqs)]

fnRs <- fastqs[grepl("_R2", fastqs)]

sample.names <- sapply(strsplit(fnFs, "_"), `[`, 1)

fnFs <- paste0(path, fnFs)

fnRs <- paste0(path, fnRs)

Plot quality score heat maps:

plotQualityProfile(fnFs[[1]])

plotQualityProfile(fnRs[[1]])

Trim sequences and remove low quality reads:

filtFs <- paste0(path, sample.names, "_F_filt.fastq.gz")

filtRs <- paste0(path, sample.names, "_R_filt.fastq.gz")

for(i in seq_along(fnFs)) {fastqPairedFilter(c(fnFs[i], fnRs[i]), c(filtFs[i], filtRs[i]), trimLeft=c(10, 10), truncLen=c(240,160), maxN=0, maxEE=2, truncQ=2, compress=TRUE, verbose=TRUE)}

Dereplicate the sequences:

derepFs <- derepFastq(filtFs, verbose=TRUE)

derepRs <- derepFastq(filtRs, verbose=TRUE)

Run the dada algorithm:

names(derepFs) <- sample.names

names(derepRs) <- sample.names

dadaRs <- dada(derepRs, err=inflateErr(tperr1,3), selfConsist = TRUE, multithread = TRUE, MAX_CONSIST = 20, pool = TRUE)

dada2:::checkConvergence(dadaRs[[1]])

dadaFs <- dada(derepFs, err=inflateErr(tperr1,3), selfConsist = TRUE, multithread = TRUE, MAX_CONSIST = 20, pool = TRUE)

dada2:::checkConvergence(dadaFs[[1]])

Make error plots:

plotErrors(dadaFs[[1]], nominalQ=TRUE)

plotErrors(dadaRs[[1]], nominalQ=TRUE)

Merge forward and reverse reads and prepare data matrix:

mergers <- mergePairs(dadaFs, derepFs, dadaRs, derepRs, verbose=TRUE)

head(mergers[[1]])

seqtab <- makeSequenceTable(mergers[names(mergers)])

dim(seqtab)

# [1] 12 4898

table(nchar(colnames(seqtab)))

# 230 231 232 233 234 235 238 239 240 242 243 244 245 246 247 251 253

# 89 4518 256 12 3 3 5 1 1 2 1 1 1 1 2 1 1

Remove chimeric sequences:

seqtab.nochim <- removeBimeraDenovo(seqtab, verbose=TRUE)

# Identified 1206 bimeras out of 4898 input sequences.

dim(seqtab.nochim)

# [1] 12 3692

sum(seqtab.nochim)/sum(seqtab)

# [1] 0.8522881

# 14.8 % of the sequences were found to be chimeric.

table(nchar(colnames(seqtab.nochim)))

# 230 231 232 233 234 235 238 239 240 242 243 244 245 246 247 251 253

# 83 3336 238 12 3 3 5 1 1 2 1 1 1 1 2 1 1

Classify sequences according to the SILVA reference:

taxa.SILVA <- assignTaxonomy(seqtab.nochim, "silva_nr_v123_train_set.fa.gz", minBoot = 70, verbose = TRUE)

colnames(taxa.SILVA) <- c("Domain", "Phylum", "Classis", "Ordo", "Familia", "Genus")

Export the results:

write.table(taxa.SILVA, file = "taxa.SILVA.txt", sep = "\t", quote = FALSE)

write.table(seqtab.nochim, file = "sequnece_variant_matrix.txt", sep = "\t", quote = FALSE)

Calculating diversity indices in R:

Input data matrices were prepared from the DADA2 output in Excel.

library(vegan); packageVersion("vegan")

# [1] ‘2.4.0’

Barley_matrix_for_diversity=as.matrix(read.table("Barley_matrix_for_diversity.txt", header=TRUE, sep="\t", row.names=1))

dim(Barley_matrix_for_diversity)

# [1] 12 3618

Barley_matrix_rel <- decostand(Barley_matrix_for_diversity, "total", MARGIN = 1)

simpson <- diversity(Barley_matrix_rel, index = "simpson")

shannon <- diversity(Barley_matrix_rel, index = "shannon")

ANOSIM in R:

Rare sequence variants containing less than 91 sequences were removed, then all zeroes in the data matrix were replaced with 0.1 to allow applying centered log-ratio transformation.

library(compositions); packageVersion("compositions")

# [1] ‘1.40.1’

Barley_matrix_for_ANOSIM =as.matrix(read.table("Barley_matrix_for_ANOSIM.txt", header=TRUE, sep="\t", row.names=1))

dim(Barley_matrix_for_ANOSIM)

# [1] 12 1074

Barley_matrix_clr=clr(Barley_matrix_for_ANOSIM)

write.table(Barley_matrix_clr, file=" Barley_matrix_clr.txt",sep="\t",quote=FALSE)

# Open the matrix in Excel and correct the shifted column heads.

Barley_matrix_clr=as.matrix(read.table("Barley_ matrix_clr.txt", header=TRUE, sep="\t", row.names=1))

dim(Barley_matrix_clr)

# [1] 12 1074

Barley_groups=as.matrix(read.table("Barley_groups.txt", header=TRUE, sep="\t", row.names=1))

Barley_groups2=data.frame(Barley_groups)

attach(Barley_groups2)

library(vegan); packageVersion("vegan")

# [1] ‘2.4.0’

Barley_ANOSIM <- anosim(Barley_matrix_clr, Treatment, permutations = 999, distance = "euclidean")

ALDEx2 in R:

Barley_matrix_for_ALDEx2=as.matrix(read.table("Barley_matrix_for_ALDEx2.txt", header=TRUE, sep="\t", row.names=1))

Barley_ matrix_for_ALDEx2_dataframe <- data.frame(Barley_matrix_for_ALDEx2)

dim(Barley_ matrix_for_ALDEx2_dataframe)

# [1] 3618 12

ALDEx2_groups <- c(rep("ambient", 6), rep("elevated", 6))

library(ALDEx2); packageVersion("ALDEx2")

# [1] ‘1.4.0’

Barley_ALDEx2 <- aldex(Barley_ matrix_for_ALDEx2_dataframe, ALDEx2_groups, mc.samples = 1000, test = "t", effect = TRUE, include.sample.summary = TRUE, verbose = TRUE)

write.table(Barley_ALDEx2, file = "Barley_ALDEx2_results.txt", sep = "\t", quote = FALSE)

aldex.plot(Barley_ALDEx2, type = "MW", test = "welch")

aldex.plot(Barley_ALDEx2, type = "MA", test = "welch")

ALDEx2 to identify taxa with differential abundance:

The fasta and count_table files were created from the DADA2 output in Excel.

Group SVs according to their taxonomic classification in mothur:

classify.seqs(fasta=Barley.fasta, count=Barley.count_table, reference=silva.nr_v123.align, taxonomy=silva.nr_v123.tax, cutoff=70)

phylotype(taxonomy=Barley.nr_v123.wang.taxonomy)

make.shared(list=Barley.nr_v123.wang.tx.list, count=Barley.count_table)

classify.otu(list=Barley.nr_v123.wang.tx.list, count=Barley.count_table, taxonomy=Barley.nr_v123.wang.taxonomy)

Format the output files for ALDEx2 in Excel.

Run ALDEx2 in R with the results from each taxonomic level

SV-sharing network

The DADA2 output was changed into mothur’s shared file format in Excel. The mapping file was made manually.

Rarefying the dataset and converting it into biom format in mothur:

sub.sample(shared=Barley.shared, size=59400)

make.biom(shared=Barley.subsample.shared)

Calculate SV-sharing network in QIIME virtual box:

make_otu_network.py -i Barley.biom -m Barley_mapping.txt -b Treatment -o Barley_SV_network

Visualize the network in Cytoscape 2.8.3 following the QIIME “Making Cytoscape Networks” tutorial: <http://qiime.org/tutorials/making_cytoscape_networks.html>

Sequence and SV numbers

| Sample | # of sequences | # of sequences after quality filtering | # of SVs |
| --- | --- | --- | --- |
| ambient 1 | 142447 | 90691 | 2830 |
| ambient 2 | 137375 | 76503 | 2931 |
| ambient 3 | 122107 | 71659 | 2535 |
| ambient 4 | 132738 | 75497 | 2686 |
| ambient 5 | 126558 | 76757 | 2731 |
| ambient 6 | 127317 | 73581 | 2911 |
| elevated 1 | 129389 | 82234 | 2708 |
| elevated 2 | 111750 | 66886 | 2583 |
| elevated 3 | 125709 | 78667 | 2363 |
| elevated 4 | 122366 | 78655 | 2288 |
| elevated 5 | 127566 | 80428 | 2737 |
| elevated 6 | 106764 | 59400 | 2659 |
